# The Nature and Treatment of Gout

**Published:** 1846-01

**Authors:** 


### ART. XIX.

*The Nature and Treatment of Gout.* By WILLIAM HENRY ROBERTSON, M.D. Physician to the Buxton Bath Charity. *London,* 1845. 8vo, pp. 372.

IT seems to us, that in writing this treatise Dr. Robertson has had it in view *to make a book,* and with this we do not altogether find fault. Mean motives (we use not the word in an offensive sense) have produced excellent ends; an interest the most personal has often swayed a man to an enterprise, a course of action, which, as regards others, has all the effects which philanthropy itself would desire to accomplish. The wish that Dr. Robertson as Physician to the Buxton Bath Charity, may feel to render his name noted in association with the successful treatment of gout, is at once natural and laudable; and to this, as already stated, we have nothing to object. We could have wished, however, that the author, in attaining his end, had inflicted on the public a less plethoric volume than he has done. Why! if gout consists in redundant, unassimilated, unreduced humours, then is Dr. Robertson's volume congenitally gouty. It is tumid with undigested extraneous matters, and if it should be born again (we mean in the way of a second edition) we should recommend that the author, before his second literary accouchement, should, by something of the nature of intellectual phlebotomy, practised on himself, lessen the chance of his again giving birth to a production, the component parts of which, even were they all sound, must at least be owned to be monstrously developed.

We do not say that Dr. Robertson's book is a stupid one, or without some useful matters; what we assert is, that in proportion to such useful matters, its bulk is unnecessarily great, and that too much of the work is occupied with diffuse, desultory observations.

The treatise is divided into eight chapters, the first of which is introductory, and the succeeding ones are devoted to a consideration of the causes, nature, history, and treatment of gout; "the treatment and management of the gouty habit of body," (chapter 7,) and the means of preventing the gout.

The first or introductory chapter presents nothing of novelty or interest. In the second chapter, or that in which the remote causes and predisposing cause are considered, we have a simple recapitulation of what is to be met with in every treatise on the subject of gout. "Hereditary influence" is stated to be the "principal remote cause." Sedentary habits, undue exercise of mind, intemperance in food, &c., are successively and prolixly discussed. The exciting causes are more summarily noticed. These are stated to be sudden changes of temperature, "an immoderately large and heavy meal," an excessive use of wine, &c. The nature of gout is next considered. It is stated to consist (p. 69.) "in the deposition of lithic (uric) acid and its compounds, with alkalies, and principally with soda, in the fibrous tissues."

The lithic acid diathesis, according to the author, presents itself under two forms, or rather has two modes of manifesting itself. In the one, the acid and its compounds escape in the urine; in the other, they are deposited in the tissues above named. The author accordingly seems to consider gout, or the gouty diathesis, essentially to consist in a morbid tendency to the deposition of uric acid or its combinations, and in a "much disordered condition of the tissue in which it is formed." (p. 72.) Dr. Robertson thinks it not improbable that the acid, though, as constituting gouty concretions, or chalk-stones, always found in a combined state, is originally deposited pure, and gradually afterwards takes soda from the blood. "This," he observes, p. 73, "would serve to explain the immediate deposition of lithic acid, where it is formed, and likwise that other alkalies besides soda are often found combined with it, although in comparatively small proportions, soda being the principal alkali of the system." He elsewhere re-states this view, (p. 78,) and expresses it as his opinion that the irritation of the capillaries of the fibrous tissues, caused by "the throwing down of crystalline particles of lithic acid, "or of its compounds with alkali," may be the true cause of gouty inflammation.

This deposition of lithic acid has been by some ascribed to "defective oxygenation of the organic atoms which have served their purpose in the economy," and which require an additional proportion of oxygen in order to form them into a soluble product capable of being eliminated as urea. The author, with some plausibility, argues, that if this view were true, gout should be most found where food containing much carbon was used, and least where such diet was least employed; but we know the contrary of this to be the case.

The chapter, on the history of gout, contains a review both of the symptoms of an arthritic attack, and also of the derangements of other organs, which accompany it. The liver is usually much disordered in its action, and the kidneys equally or more so. We are, however, glad to hear Dr. Robertson bearing witness to a fact we have ourselves sometimes observed, namely, the presence of gout without derangement of the renal secretion. There has been an endeavour of late to exalt the indications afforded by the urine, even beyond their rank, high as that may be allowed to be, and to exaggerate the frequency with which these indications may be detected. "In some of the worst cases of gout," says Dr. Robertson,

(p. 130,) "I have met with, the urine was never deranged in quantity or character, either during or between the paroxysms." He adds, however, that in these cases, the hepatic, or some other functions, were usually disordered.

The usual deposits in an attack of gout are, in the acute stage, lithic acid and lithates of ammonia and soda. Phosphatic deposits may occur after some time, more especially if the patient's constitution be shattered. These consist chiefly of the phosphate of lime and of magnesia.

In regard to therapeutical indications afforded by the states of the urine, the author makes an observation (p. 133) in keeping with the one we have just quoted above, and the accuracy of which we can confirm, and which we cite as calculated to check the disposition prevalent with the class we may call urinary pathologists, too much to found treatment on a single branch of symptomatology, and not, as true physicians ought to do, on a comprehensive survey of the aggregate of morbid phenomena. "As to the treatment of gout," says Dr. Robertson, "it will be found that the condition of the urine, judged of *per se*, has little to do in regulating or modifying it, however valuable it may sometimes be, when looked upon as subsidiary to other treatment."

We cannot accompany the author through his long and (if condensed and better methodized) really valuable history of gout. The fault we have to find with this portion of the work is this, that the author, by noticing in connexion with gout, almost every symptom of derangement incident to the human body, and the occasional association of which with arthritic disease may be termed casual, destroys, as it were, the *individuality* of the malady of which his work specially treats, and converts his book into a sort of confused jumble of universal pathology.

The treatment recommended by the author, is, on the whole, judicious, and is, in short, that which is usually pursued. Leeching, he observes, as well as the lancet, or cupping, are seldom of the slightest use in simple gout: they may do harm, but do no good. Purgatives and diaphoretics, he states, to be the evacuants chiefly to be depended on; and he might have added diuretics, a free action of the kidney being nearly as indispensable as a free action of the bowels. The hostility of Sydenham to purgatives, both during the gouty paroxysm and the interval, is well known. The author does not entirely adopt on this subject the opinion of the great authority just named; neither do we. In a multitude of cases of gout, both during the paroxysms and at intervening periods, judicious regulations of the bowels, we do not say violent purging, is of evident use, as far as our observation extends. The purgatives must not, indeed, be cold and drastic, but warm, cordial, and what is called stomachic. The author justly discountenances the addition of narcotics to purgative medicines: the union is at once irrational, useless, and pernicious. Colchicum, so far as it is a narcotic, is to be excepted from this remark. We have not yet brought ourselves to regard it as a narcotic at all, but attribute the abatement of pain, which it often affords, both in gout and rheumatism, to its setting suspended secretions free, as for example, the biliary and the urinary. Its lowering of the heart's action we refer to the same cause.

We shall not follow the author through the remaining part of his volume, in which, had brevity and the omission of speculative discussion but been more attended to, the useful practical matter, now made to appear only *longis intervallis*, would have presented itself to much more advantage, and would have been more conveniently and readily got at by the reader. He finishes his volume by two chapters, the one of which is devoted to the consideration of the treatment and management of the gouty habit; and the other to the prevention of gout. Both of these contain no small amount of extremely useful and intelligent hints, directions, and suggestions, and are less chargeable with unnecessary prolixity than the earlier portions of the work.

Our opinion of Dr. Robertson's treatise may be gathered from our preceding remarks. It is this; that within the 372 pages constituting his volume, there is matter which, if properly condensed and arranged, would form a very useful practical volume of 120 pages, that is, one third the size of the book actually before us. In other words, we think two thirds of the volume might with advantage be spared to us. At the same time we cannot conclude without expressing our opinion that the author is a man of very considerable intelligence; and many even of the "faults" of his volume "lean" to "the side" of ability.

---

